# Draft Genome Sequences of a Novel Lineage of *Armatimonadetes* Recovered from Japanese Hot Springs

**DOI:** 10.1128/genomeA.00820-17

**Published:** 2017-10-05

**Authors:** Lewis M. Ward, Shawn E. McGlynn, Woodward W. Fischer

**Affiliations:** a Division of Geological and Planetary Sciences, California Institute of Technology, Pasadena, California, USA; b Earth-Life Science Institute, Tokyo Institute of Technology, Meguro-ko, Japan

## Abstract

Here, we report two draft genome sequences from a novel lineage within the *Armatimonadetes* phylum recovered from metagenomes sequenced from Japanese hot spring microbial mats. These organisms are aerobic and represent a new lineage related to the characterized *Chthonomonas* and *Fimbriimonas* groups, and they expand the diversity of this enigmatic phylum.

## GENOME ANNOUNCEMENT

The *Armatimonadetes* phylum (i.e., OP10) has only four isolates and is primarily known from environmental sequences (1). Here, we describe two closely related draft genome sequences of a novel lineage of *Armatimonadetes* recovered from Japanese hot spring metagenomes.

These genomes were recovered as part of a study of Japanese hot spring microbial mats (2). CP1_7O was recovered from sequencing of a cone-forming microbial mat at Nakabusa Onsen in Nagano Prefecture. This spring is sulfidic and alkaline, and was 48°C with pH 8.3 at the point of sampling. JP3_11 was recovered from a microbial mat sample from an iron-rich intertidal hot spring at Jinata Onsen, Tokyo Prefecture. The spring was 46°C and pH 6.5 at the location of sampling.

Samples (∼0.25 cm^3^) were collected using sterile forceps. DNA was preserved in the field following cell lysis using Zymo TerraLyzer BashingBead matrix and Xpedition lysis buffer. Cells were disrupted by attaching sample tubes to the blade of a cordless reciprocating saw and operating for 1 min. DNA was purified with a Zymo soil/fecal DNA extraction kit (Zymo Research, Irvine, CA) and quantified with a Qubit 3.0 fluorimeter (Life Technologies, Inc., Carlsbad, CA). DNA was submitted to SeqMatic LLC (Fremont, CA) for library preparation and sequencing by Illumina HiSeq. Sequences were assembled with MegaHit version 1.02 (3) and binned using MetaWatt version 3.5.2 (4). Completeness and contamination of bins were estimated using CheckM (5).

The CP1_7O draft genome is 2.45 Mb, has 59.7% GC content, is made up of 470 contigs, and contains 2,176 coding sequences and 47 RNAs. The JP3_11 genome is 2.60 Mb, has 59.4% GC content, is made up of 359 contigs, and contains 2,313 coding sequences and 50 RNAs. Based on an analysis of the single-copy marker genes by CheckM, the CP1_7O draft genome is estimated to be 91.67% complete, and that of JP3_11 is estimated to be 89.35% complete. These genomes contain identical 16S genes; the most similar 16S sequence to those from a cultured organism is only 82% identity to *Acidothermus cellulolyticus*.

Both genomes described here contain genes for aerobic respiration, including alternative complex III (ACIII), an A family heme copper oxidoreductase (HCO), and a B family HCO. The phylogeny of ACIII and B family HCO genes in the *Armatimonadetes* and closely related *Chloroflexi* largely concur with the organismal tree based on conserved ribosomal proteins; consequently, aerobic respiration may represent a vertically inherited ancestral metabolic trait. While members of the *Chloroflexi* lack an outer membrane and associated genes, such as those involved in lipopolysaccharide synthesis (e.g., reference 6, and L. M. Ward, J. Hemp, P. M. Shih, S. E. McGlynn, and W. W. Fischer, unpublished data), CP1_7O and JP3_11 contain genes coding for lipopolysaccharide (LPS) synthesis and outer membrane proteins, such as BamA. This suggests that these *Armatimonadetes* are diderm, and that the unusual membrane architecture of the *Chloroflexi* phylum may be a derived trait, a pattern consistent with recent observations from the *Firmicutes* (7).

### Accession number(s).

This whole-genome shotgun project was deposited in DDBJ/EMBL/GenBank under the accession numbers NKPU00000000 (CP1_7O) and NKPV00000000 (JP3_11).
